# Multidrug-Resistant, Including Extended-Spectrum Beta Lactamase-Producing and Quinolone-Resistant, *Escherichia coli* Isolated from Poultry and Domestic Pigs in Dar es Salaam, Tanzania

**DOI:** 10.3390/antibiotics10040406

**Published:** 2021-04-09

**Authors:** Zuhura I. Kimera, Fauster X. Mgaya, Gerald Misinzo, Stephen E. Mshana, Nyambura Moremi, Mecky I. N. Matee

**Affiliations:** 1 Department of Microbiology and Immunology, School of Medicine, Muhimbili University of Health and Allied Sciences, Dar es Salaam 11103, Tanzania; fauster.mgaya@sacids.org (F.X.M.); mecky.matee@sacids.org (M.I.N.M.); 2 Ministry of Livestock and Fisheries, Dodoma 40487, Tanzania; 3 SACIDS Africa Centre of Excellence for Infectious Diseases, Sokoine University of Agriculture, Morogoro 67125, Tanzania; gerald.misinzo@sacids.org; 4 Department of Veterinary Microbiology, Parasitology and Biotechnology, College of Veterinary Medicine and Biomedical Sciences, Sokoine University of Agriculture, Morogoro 67125, Tanzania; 5 Department of Microbiology and Immunology, School of Medicine, Catholic University of Health and Allied Sciences, Mwanza 33109, Tanzania; stephen.mshana@sacids.org; 6 Ministry of Health, Community Development, Gender, Elderly and Children, Dar es Salaam 11101, Tanzania; nyambura.moremi@afya.go.tz

**Keywords:** poultry, domestic pigs, antibiotics, antimicrobial resistance, Msimbazi basin, farmers, *Enterobacteriaceae*

## Abstract

We determined the phenotypic profile of multidrug-resistant (MDR) *Escherichia coli* isolated from 698 samples (390 and 308 from poultry and domestic pigs, respectively). In total, 562 *Enterobacteria* were isolated. About 80.5% of the isolates were *E. coli*. Occurrence of *E. coli* was significantly higher among domestic pigs (73.1%) than in poultry (60.5%) (*p* = 0.000). In both poultry and domestic pigs, *E. coli* isolates were highly resistant to tetracycline (63.5%), nalidixic acid (53.7%), ampicillin (52.3%), and trimethoprim/sulfamethoxazole (50.9%). About 51.6%, 65.3%, and 53.7% of *E. coli* were MDR, extended-spectrum beta lactamase-producing *enterobacteriaceae* (ESBL-PE), and quinolone-resistant, respectively. A total of 68% of the extended-spectrum beta lactamase (ESBL) producers were also resistant to quinolones. For all tested antibiotics, resistance was significantly higher in ESBL-producing and quinolone-resistant isolates than the non-ESBL producers and non-quinolone-resistant *E. coli*. Eight isolates were resistant to eight classes of antimicrobials. We compared phenotypic with genotypic results of 20 MDR *E. coli* isolates, ESBL producers, and quinolone-resistant strains and found 80% harbored *bla*CTX-M, 15% *aac*(*6*)-*lb*-*cr*, 10% *qnrB*, and 5% *qepA*. None harbored TEM, SHV, *qnrA*, *qnrS*, *qnrC*, or *qnrD*. The observed pattern and level of resistance render this portfolio of antibiotics ineffective for their intended use.

## 1. Introduction

Antimicrobial resistance (AMR) is a complex global matter, which requires concerted efforts to curb its serious consequences [1]. Globally, it is estimated that 10 million people will die annually by 2050 if no appropriate measures are taken [2]. It is projected, by that time, that AMR will be the leading cause of death worldwide [3]. This has prompted research on natural products such as ascidians that have shown broad antimicrobial (antibacterial, antifungal, and antiviral) activity [4]. The emergence and spread of AMR genes have been largely associated with human activities such as inappropriate use of antimicrobials in human healthcare, crop and animal farming, and weak infection prevention and control practices/biosecurity in both human and animal healthcare facilities [5]. Globally, in 2014, the consumption of antibiotics for human use was estimated to be 70 billion standard units/year, while for livestock, the amount was approximately 63,151 tons/year [6]. It is predicted that by 2025, this amount will increase by 30% and 67% for humans and animals, respectively [7]. Global trends in sales of antimicrobials for animal production show an 11.5% rise by 2030, mainly due to increased demand in animal proteins [8]. Other studies have estimated that 73% of all antimicrobials sold in the world are used in animal production [9].

AMR in humans has been associated with high morbidity and mortality rates, especially in low-income countries, due to an inability to detect resistance and limited treatment options [10,11,12]. In Tanzania, infections with AMR and multidrug-resistant (MDR) bacteria, especially extended-spectrum beta lactamase (ESBL) and carbapenemase producers, have been associated with increased morbidity and mortality [13,14,15]. Some of the studies conducted in the country have associated AMR with inappropriate prescriptions and self-medication [16,17], and often antibiotics are dispensed without a prescription [18]. In animals, AMR, which is largely due to intensive farming associated with over-use of antimicrobial agents, has been shown to contribute to reduced productivity and economic crisis, as well as spreading resistance organisms to humans and the environment and causing infections that are hard to treat [19,20].

In Tanzania, the demand for short-cycle animal stocks is expected to increase sharply within a short period. For example, pork consumption has been projected to increase from 42.7 thousand to 170 thousand metric tons from 2017 to 2030 [21]. Likewise, estimates show that annual chicken meat production will increase from 22,000 tons in 2017 to 37,200 tons in 2022 [21]. This increased demand has led to intensified farming systems and increased use of antimicrobials, including antibiotics [22,23]. In a recent study conducted among poultry and pig farmers in the Msimbazi River basin, we found high usage of veterinary antimicrobials mainly for prophylaxis (87.6%) compared to therapy (80.5%) [24]. Unfortunately, regulation of antibiotic use in animal food production in Tanzania faces several challenges such as weak regulation in the use of antimicrobials, weak surveillance systems, the tendency for animal owners to stock drugs, engaging unskilled people to treat animals, and a high degree of drug abuse by livestock keepers [25,26,27]. Not surprisingly, antibiotic-resistant bacteria have been reported in about three quarters of food animals, mainly in rural and suburban areas [28,29,30,31]. Food animals carrying AMR organisms can affect human health [32] and eventually contaminate the environment [33], and therefore monitoring the magnitude and pattern of resistance in them is essential in curbing the spread of resistant genes [34].

We deliberately conducted this study in the Msimbazi River basin, a unique ecosystem and the most densely populated area in Tanzania, which supplies most of the poultry, eggs, and domestic pigs for the city of Dar es Salaam [35,36,37]. The aim was to determine phenotypic AMR profiles of MDR *Escherichia coli* in domestic pigs and poultry. *E. coli* harbor mobile genetic elements such as plasmids and transposons which facilitates the rapid spread of resistance genes from animals to humans via the environment [38]. Our focus was on genes encoding for ESBL production and quinolone resistance, responsible for resisting the most frequently used antibiotics in animal production and in the treatment of human infections in Tanzania and many African countries [19,24,39].

## 2. Results

### 2.1. Detection of Enterobacteriaceae Isolates, Prevalence of Resistance, and Comparative Analysis of Antibiotic-Resistant Profiles from Poultry and Domestic Pig Samples

As shown in Table 1, a total of 562 *Enterobacteriaceae* isolates were obtained from 698 samples (390 and 308 from poultry and domestic pigs, respectively). About 80.5% of the isolates were *E. coli*. Isolation of *E. coli* was higher in domestic pigs (73.1%) than in poultry (60.5%) (*p* = 0.000), while poultry harbored more *K. pneumoniae* (2.3%) compared to domestic pigs (1.9%). Other *Enterobacteriaceae* detected were *Klebsiella oxytoca*, *Pantoea species*, *Leclercia adercarboxylate*, *Citrobacter species*, *Erwinia species*, *Serratia odorifera*, and *Salmonella enterica*.

**Figure 1:**
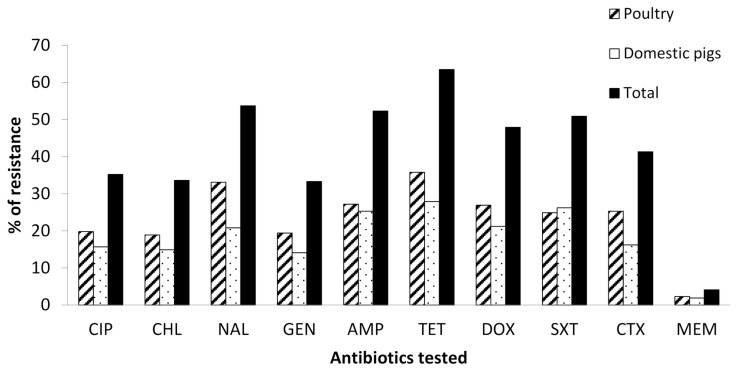
Percentage of antibiotic resistance from the poultry and domestic pig isolates. Key: CIP, ciprofloxacin; CHL, chloramphenicol; NAL, nalidixic acid; GEN, gentamycin; AMP, ampicillin; TET, tetracycline; DOX, doxycycline; SXT, trimethoprim/sulfamethoxazole; CTX, cefotaxime; MEM, meropenem.

Overall, the highest percentage of resistance in both poultry and domestic pigs was for tetracycline (63.5%), followed by nalidixic acid (53.7%), ampicillin (52.3%), and trimethoprim/sulfamethoxazole (50.9%). As shown in Figure 1 and Table 2, poultry harbored more resistant isolates (55.2%) to almost all tested antibiotics compared to domestic pigs (44.8%). The resistances against nalidixic acid (*p* = 0.006), trimethoprim/sulfamethoxazole (*p* = 0.005), and cefotaxime (*p* = 0.016) were significantly different between poultry and domestic pigs.

As shown in Table 3, isolates from poultry showed no significant variations in resistance by location against all of the tested antibiotics except for gentamycin (*p* = 0.046), ampicillin (*p* = 0.026), and doxycycline (*p* = 0.018). For domestic pigs, there were significant variations by location in resistance against all the tested drugs except for doxycycline (*p* = 0.101) (Table 4).

### 2.2. Multidrug Resistance of E. coli Isolates in Cloacal and Rectal Swabs from Poultry and Domestic Pigs

Table 5 shows that out of 461 *E. coli* isolates, 51.6% (*n* = 238) were MDR against the tested drugs. The most common resistance patterns observed were QNL/PHE/AMN/PEN/TET/SUL/CEP (62 isolates), QNL/PHE/AMN/PEN/TET/SUL (20 isolates), QNL/PHE/PEN/TET/SUL (16 isolates), QNL/PEN/TET/SUL (15 isolates), and PEN/TET/SUL (14 isolates). Eight isolates were resistant to eight classes of antimicrobials.

### 2.3. ESBL-Producing E. coli Isolated from the Cloacal and Rectal Swabs from Poultry and Domestic Pigs

From the 461 *E. coli* isolates of cloacal and rectal swabs screened for ESBL production using 2 µg/mL cefotaxime, 65.3% (301/461) were positive, and of these positive isolates, all were confirmed to be extended-spectrum beta lactamase-producing *Enterobacteriaceae* (ESBL-PE). The ESBL isolates were significantly more resistant to tetracycline, CIP, doxycycline, trimethoprim/sulfamethoxazole, and nalidixic acid than non-ESBL producers (Table 6).

### 2.4. Quinolone-Resistant E. coli in Cloacal and Rectal Swabs from Poultry and Domestic Pigs

Out of 461 *E. coli* isolates tested for quinolone resistance, 37.5% (*n* = 173) were found to be quinolone-resistant [40] and were significantly more resistant to all the tested antibiotics compared with non-quinolone isolates (Table 7). As shown in Table 8, both ESBL producers and quinolone resistance depicted the various level of resistance, with tetracycline receiving significant resistance both in ESBL and quinolone-resistant isolates. About 68.1% of ESBL producers were resistant to quinolone. Quinolone resistance significantly predicted higher resistance to CIP, CHL, GEN, TET, and SXT compared to the ESBL phenotype.

### 2.5. Genotypic Identification of ESBL Resistant Genes (CTX-M, TEM, SHV) and Quinolone-Resistant Genes (qnrA, qnrB, qnrS, qnrC, qnrD, aac(6′)-lb-cr, and qepA)

**Figure 2:**
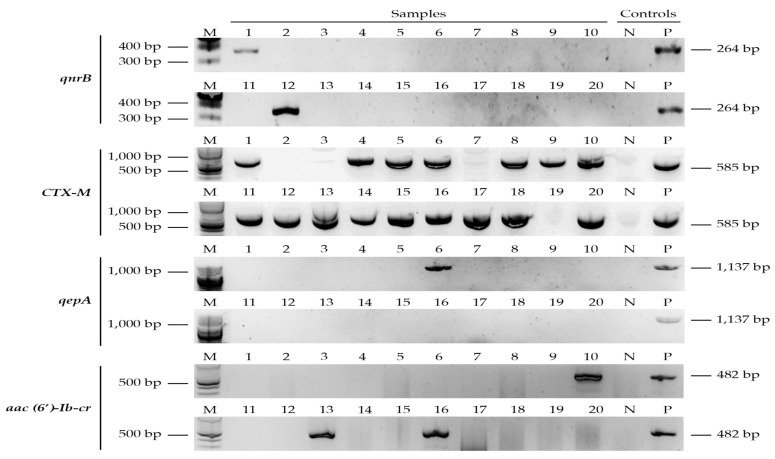
Gel electrophoretic bands of ESBL and quinolone-resistant genotypes (*qnrB*, CTX-M, *qepA*, and *aac*(*6*)-*lb*-*cr*). Letters: M—DNA ladder, N—negative control, and P—positive control. Positive samples are numbers 1, 10, 12, 13, and 16 (*qnrB*, *aac*(*6*)-*lb*-*cr*, and *qepA*) and samples 1, 4, 5, 6, 8, 9, 10, 11, 12, 13, 14, 15, 16, 17, 18, and 20 (CTX-M).

Out of 20 *E. coli* selected from MDR isolates, the CTX-M gene was detected in 16/20 (80%), while TEM and SHV were not detected. The quinolone-resistant genes (*qrnB*, *aac*(*6*)-*lb-cr*, and *qepA*) were detected in 6/20 (30%) (Table 9 and Figure 2). One isolate from the poultry harbored both *qepA* and *aac*(*6*)-*lb*-*cr* genes. The resistant genes *qnrA, qnrS*, *qnrC*, and *qnrD* were not detected in any of the tested isolates.

## 3. Discussion

In this study, 80.5% of the isolates from 698 samples of rectal and cloaca swabs from domestic pigs and poultry were recovered. The levels of resistance to the tested antibiotics were higher especially for tetracycline (65.6%), nalidixic acid (53.7%), ampicillin (52.3%), and trimethoprim/sulfamethoxazole (50.9%). Overall, 51.6%, which is close to half of all *E. coli* isolates from the poultry and domestic pigs, exhibited multidrug resistance against three to eight classes of antimicrobial agents tested, with the most resistant pattern being against QNL/PHE/AMN/PEN/TET/SUL/CEP. The level of resistance identified in this study compares with the findings reported in Zimbabwe, Nigeria, Ghana, and China which were attributed to extensive use of veterinary drugs in animal farming [41,42,43,44]. These levels of resistance in the current study were not surprising basing on the mode of animal farming that involved intensive use of veterinary drugs for therapeutics and disease prevention. Recent findings reported that the veterinary antimicrobial classes used most extensively in animal farming are tetracycline, penicillin, quinolones, and sulphonamides [19,24,39], thus leading to the higher resistance level. The findings on the levels of MDR are in line with those reported in Tanzania, Ghana, and Angola, of which 42%, 56.9%, and 50%, respectively, of the identified *E. coli* isolates from food animals were MDR [45,46,47]. However, the percentages of MDR in this study are lower than the ones reported in Ghana, Nigeria, and Zambia [43,48,49]. The possible explanation to the level of MDR in this study might certainly be due to the selection pressure and environmental contamination by a variety of wastes including plastic litter, industrial effluents, and uncontrolled disposal of human and veterinary drugs [50,51]. We found that poultry harbored isolates that were more resistant (55.2%) to almost all tested antibiotics. Previous findings from Tanzania reported that poultry farming is associated with uncontrolled use of both human and veterinary antimicrobials, mainly for growth promotion compared to therapeutics, and metaphylaxis is very commonly practiced by the majority of farmers [24,52]. The level of resistance to these tested antibiotics for isolates from poultry corresponds to previous studies [50,53,54], showing poultry farming involves intensive and extensive use of antibiotics compared to other domestic animals. Tetracycline, aminoglycosides, penicillin, quinolone, and sulphonamides are among the antibiotics reported to be commonly used in poultry in Ghana, Cameroon, and Sudan [55,56,57], leading to the development of antibiotic resistance. On the other hand, we found a low level of resistance against meropenem (less than 10%) both in poultry and domestic pigs, which is not surprising given the fact that these antibiotics are not easily accessed due to cost [58,59].

Notably, we found significant variations in antibiotic resistance by wards among isolates obtained from both poultry and domestic pigs, which may be due to variations in farming conditions and the use of antibiotics [60,61]. However, this was not investigated in this study and may therefore require further research. Nonetheless, this finding is significant, showing a lack of professional guidance on the use of antimicrobials in animal farming, supporting previous studies in the same area showing unregulated use of antibiotics that are largely obtained over the counter [24]. Farmers face several challenges that include reduced drug quality, substandard/counterfeit veterinary drugs, and uncontrolled use of drugs in healthcare, agriculture, and industrial settings, and lack of veterinary services [62,63].

In this study, the majority of the *E. coli* isolates (65.3%) were found to be ESBL producers, a level similar to other studies [34,64,65,66] but higher than levels reported in Zambia (20%), Nigeria (37.8%), and Ghana (29%) [49,67,68]. All ESBL producers were significantly more resistant to all the tested antimicrobials as compared to the non-ESBL producers, suggesting selective pressure due to extensive use of beta-lactam and cephalosporin in animal farming, and the existence of multiple resistance mechanisms resulting from indiscriminate use of veterinary drugs [69,70,71,72]. On the other hand, approximately half, 37.5%, of all isolates were found to be quinolone (ciprofloxacin or nalidixic acid)-resistant and these strains were more resistant to all the tested antibiotics compared to non-quinolone isolates, probably due to persistent use of antibiotics for prophylaxis, therapeutics, metaphylaxis, and growth promotion, a finding reported in other studies [42,73].

Notably, resistance to tetracycline and trimethoprim/sulfamethoxazole was higher both in ESBL producers and quinolone-resistant isolates, suggesting the presence of an association between ESBL producers and quinolone resistance, as previously reported [74,75]. The association between ESBL and quinolone resistance may highlight the presence of similar resistance mechanisms in the clinical and environmental setup, and intensive and prolonged use of beta-lactam drugs, cephalosporin, and quinolone drugs in poultry and domestic pig farming [76]. The findings are in line with other studies that reported the linkage between ESBL producers and quinolone-resistant genes due to co- transmission of resistant genes among members of the family *Enterobacteriaceae*, leading to dissemination of MDR organisms [70,72,74,76,77].

We compared phenotypic with genotypic results of 20 MDR *E. coli* isolates, ESBL producers, and quinolone-resistant strains and found that about 80% harbored *bla*_CTX-M_, 15% *aac*(*6*)-*Ib*-*cr*, and 5% *qepA.* None of the isolates harbored TEM, SHV, *qnrA*, *qnrS*, *qnrC*, or *qnrD*. We noted that some of the isolates that were sensitive by the phenotypic method harbored resistance genes, a phenomenon that has been observed by others [72,78]. Studies have shown that phenotypic resistance is dependent on the mode and level of gene expression [79,80]. On the other hand, some isolates that were phenotypically resistant did not harbor the screened genes, implying that other genes such as *ampC*, VEB, OXA, PER, *oqxA*, or *oqxB* might be responsible [74,81].

The high levels of AMR associated with poultry and domestic pig farming present a risk to human health presented by the ineffectiveness of the currently used antibiotics [62,66,82], thus causing human and animal infections that are difficult to treat [50,55,83]. This study has some limitations.

Other *Enterobacteria* spp. isolates were too few for detailed subanalysis; therefore, we decided to focus on *E. coli*. Secondly, we did not perform molecular characterization of all of the resistant isolates, which may be considered as a limitation. However, the phenotypic analysis conducted was based on the internationally recognized and standardized Clinical Laboratory Standard Institute (CLSI) 2019 protocol and guidelines [84], thus reflecting the real magnitude and pattern of AMR in the study setting. Nonetheless, we are planning to collect isolates from humans and the environment and perform genotypic studies in order to understand the flow of resistomes across the human, animal, and environment compartments. Lastly, we could not analyze samples coming from poultry and domestic pigs not treated with antibiotics as we could not find such farms due to the widespread use of antimicrobials in animal production in this community [19,26,39].

## 4. Methodology

### 4.1. Selection of Study Farms and Animals

This study was conducted in Kinondoni, Kisarawe, and Ilala districts that form part of the Msimbazi River basin. Eight wards were included, namely, Kisarawe, Pugu station, Gongolamboto, Ukonga, Kipawa, Segerea, Kinyerezi, and Buguruni. Poultry farms were selected based on having more than 100 broilers and/or layers and 50 or more improved local chickens kept for commercial purposes. For each poultry farm, 5% out of 100 flocks were sampled. Animals of less than two weeks were not sampled under the assumption that they were at the early stage of production. The selection of pig farms was based on the herd having animals that were ready to be slaughtered in which 10% of the animals were selected. Poultry and pig farms included in this study were randomly selected from a list provided by the ward livestock officers within the study area.

### 4.2. Sample Collection

About 1 g of fecal materials from the cloaca and rectum from poultry and domestic pigs was collected aseptically using a sterile cotton swab (Himedia, Mumbai, India). The cotton swabs were then placed into the sterile tube filled with 3 mL Cary Blair medium (Oxoid, Basingstoke, UK). All samples were transported in a cool box containing ice packs at a temperature of 2 to 8 °C and processed within 2 h of collection in the Microbiology Teaching Laboratory at the Muhimbili University and Allied Sciences (MUHAS).

### 4.3. Isolation of Bacteria

Specimens from cloacal/rectal swabs were directly streaked onto the MacConkey agar (Oxoid, Basingstoke, UK) without antibiotics and incubated at 37 °C aerobically for 24 h. A single colony from predominant morphologically similar colonies was picked from each plain MacConkey agar plate and subcultured in a nutrient agar (Hi media, Mumbai, India). Colonies on nutrient agar were identified by colonial morphology, Gram stain, catalase and oxidase production [85], and various biochemical tests (indole, methyl red, Voges–Proskauer, and citrate utilization test) and were later confirmed by API 20E following the manufacturer’s recommendations (BioMérieux, Marcyl’Etoile, France) [86]. Briefly, a single colony was emulsified into sterile saline and filled in the compartments and then incubated at 37 °C for 18 to 24 h aerobically in a wet chamber of analytical profile index (API), API 20E strips (BioMérieux, Marcy-Etoile, France). *E. coli* and other *Enterobacteriaceae* were identified at the species level.

### 4.4. Antibiotic Susceptibility Testing

The antimicrobial susceptibility testing was conducted using the Kirby–Bauer disc diffusion method on Mueller Hinton Agar (Becton, Dickinson and Company, MD, USA) based on the CLSI standards [76]. Antibiotics tested were doxycycline (30 µg), cefotaxime (30 µg), nalidixic acid (30 µg), ciprofloxacin (5 µg), ampicillin (10µg), tetracycline (30 µg), chloramphenicol (30 µg), gentamicin (10 µg), meropenem (30 µg), and trimethoprim/sulfamethoxazole (1.25 µg/23.75 µg). These antibiotics are considered by the World Health Organization (WHO) to be clinical and useful in animal production [87]. One to two colonies from the pure culture of the identified lactose fermenters were emulsified into 5 mL of sterile saline. The suspensions were adjusted to achieve turbidity equivalent to 0.5 McFarland standard solutions [84], emulsified using sterile cotton swabs onto Mueller Hinton Agar plate, and incubated at 37 °C for 16 to 18 h. The inhibition zone of each antimicrobial agent was measured after 16 to 18 hours’ incubation. Results were interpreted according to the CLSI standards, and *E. coli* strain American Type Culture Collection (ATCC) 29522 and *K. pneumoniae* strain ATCC 700603 were used as controls. An isolate was considered to be multidrug-resistant (MDR) if it was non-susceptible to three or more drugs from different classes of antimicrobial [88].

### 4.5. Screening for ESBL

The confirmed *E. coli* isolates (by API 20E strips) from plain MacConkey agar were inoculated onto MacConkey agar containing 2 µg/mL cefotaxime for preliminary screening of ESBL production [89]. Confirmation of ESBL production was conducted using the combination disk diffusion method, with cefotaxime (30 µg) alone and in combination with clavulanic acid (10 µg), and with ceftazidime (30 µg) alone and combination with clavulanic acid (10 µg), and a zone of inhibition of more than or equal to 5 mm confirmed ESBL production. *K. pneumoniae* (ATCC 700603) was used as a positive control (ESBL-positive strain) and *E. coli* (ATCC 25922) used as the ESBL-negative strain, and results were interpreted as per CLSI standards 2019.

### 4.6. Polymerase Chain Reaction (PCR)

#### 4.6.1. DNA Extraction

ESBL-producing *E. coli* isolates were inoculated on nutrient agar and incubated aerobically at 37 °C for 24 h. DNA was extracted by boiling in a water bath at 100 °C for 10 min, followed by centrifugation at 1500 rpm for 3 min. The supernatant containing DNA was transferred into a sterile Eppendorf PCR tube (Eppendorf AG, Hamburg, Germany) and centrifugation and separation of supernatant were repeated three times. The concentration of DNA was determined by a Nanodrop spectrophotometer (Biochrom LTD, Cambridge, England) at 260/280 wavelength (ranging from 1.5 to 1.8). DNA was stored at −20 °C, before being used for detection of ESBL genes (CTX-M, TEM, and SHV) and PMQR genes (*qnrA*, *qnrB*, *qnrS*, *qnrC*, *qnrD*, *qepA*, and *aac*(*6*′)-*Ib*-*cr*).

The One Tag Master Mix Hot Start DNA polymerase kit (New England Biolabs, Ipswich, MA, USA) was used in detection of resistance genes. Total PCR reaction volumes were 25 µL, consisting of One Tag Master Mix 2X Standard buffer 12.5 µL, 10 µM forward primer 0.5 µL, 10 µM reverse primer 0.5 µL, nuclease-free water 9.5 µL, and DNA template 2 µL. The primers used in amplification of the respective *E. coli* resistance genes are listed in Table 10 below.

#### 4.6.2. Molecular Detection of CTX-M Genes

ESBL-producing *E. coli* isolates were screened for the CTX-M gene using a uniplex PCR-based technique [90]. PCR conditions involved initial denaturation at 96 °C for 5 min, followed by 35 cycles of denaturation at 96 °C for 30 s, annealing at 56 °C for 40 s, extension at 72 °C for 60 s, and final extension at 72 °C for 10 min.

#### 4.6.3. Detection of TEM and SHV Genes

ESBL genes *TEM* and *SHV* were screened by a uniplex PCR-based assay involving initial denaturation at 95 °C for 5 min, followed by 35 cycles of denaturation at 94 °C for 30 s, annealing at 56 °C (TEM and SHV) for 40 s, extension at 72 °C for 1 min, and final extension at 72 °C for 10 min [94].

#### 4.6.4. Detection of Quinolone-Resistant Genes (*qnrA*, *qnrB*, and *qnrS*)

The quinolone-resistant genes (*qnrA*, *qnrB*, and *qnrS*) were amplified and detected using multiplex PCR assay [75]. This involved initial denaturation at 94 °C for 5 min, followed by 32 cycles of denaturation at 94 °C for 45 s, annealing at 53 °C for 1 min, extension at 72 °C for 1 min, and final extension at 72 °C for 10 min [75].

#### 4.6.5. Detection of *aac*(*6*′)-*lb*-*cr* Gene

The *aac*(*6*′)*-lb*-*cr* gene was screened by a uniplex PCR-based assay [93] using the following amplification conditions: initial denaturation at 94 °C for 5 min, followed by 34 cycles of denaturation at 94 °C for 45 s, annealing at 55 °C for 45 s, extension at 72 °C for 45 s, and final extension at 72 °C for 10 min [93].

#### 4.6.6. Detection of PMQR Genes (*qepA*, *qnrC*, and *qnrD*)

The *qepA* gene was screened by a uniplex PCR-based assay [93] using the following amplification conditions: initial denaturation at 96 °C for 5 min, followed by 30 cycles of denaturation at 96 °C for 30 s, annealing at 56 °C for 30 s, extension at 72 °C for 60 s, and final extension at 72 °C for 5 min [75].

### 4.7. Ethical Considerations

The ethical clearance was provided by the Medical Research Coordinating Committee of the National Institute for Medical Research (NIMR) of Tanzania (Reference No. NIMR/HQ/R.8a/Vol. IX/3133), and Muhimbili University of Health and Allied Sciences (Permit No. DA.282/298/01.C). The permission was sought from the relevant authorities that are the municipal directors at the three districts.

### 4.8. Data Management

The data were entered in Excel version Office 2007 and then transferred to SPSS version 20.0 for Windows (IBM Corp, Armonk, NY, USA) software for statistical analysis. Categorical variables were described as frequencies and percentages. The chi-square test was used to determine the difference, and a *p*-value of less than 0.05 was considered significant.

## 5. Conclusions

The high levels of AMR as well as ESBL producer, quinolone-resistant, and MDR (up eight different classes) isolates associated with poultry and domestic pig farming seem to render the currently used antibiotics ineffective for their intended use, and their continued use potentially escalates the burden of antimicrobial resistance beyond these animal species.

## Figures and Tables

**Table 1:** Frequency of *Enterobacteriaceae* isolates from poultry and domestic pigs.

Organism	Isolates Recovered from Poultry (*n* = 310)		Isolates Recovered from Domestic Pigs (*n* = 252)	
	Number	Percentage	Number	Percentage
*Escherichia coli*	236	60.5	225	73.1
*Klebsiella pneumoniae*	9	2.3	6	1.9
*Klebsiella oxytoca*	5	1.3	2	0.6
*Pantoea* spp.	32	8.2	2	0.6
*Leclercia adercarboxylate*	5	1.3	1	0.3
*Citrobacter* spp.	5	1.3	1	0.3
*Kluyvera* spp.	4	1.0	4	1.3
*Erwinia* spp.	8	2.1	-	-
*Serratia odorifera*	3	0.8	5	1.6
*Salmonella enterica*	2	0.5	1	0.3

**Table 2:** Comparative analysis of antibiotic resistance of isolates from poultry versus domestic pigs.

Antibiotic	% of Resistance in Poultry (*n* = 236)	% of Resistance in Domestic Pigs (*n* = 225)	Chi-Square	*p*-Value
CIP (*n* = 199)	28.5	28.6	0.360	0.835
CHL (*n* =190)	27.2	27.3	0.360	0.835
NAL (*n* = 303)	47.3	38.0	10.090	0.006
GEN (*n* = 188)	27.7	26.0	0.845	0.655
AMP (*n* = 295)	39.0	46.4	3.997	0.136
TET (*n* = 358)	51.3	51.3	0.447	0.800
DOX (*n* 270)	38.5	39.0	0.346	0.841
SXT (*n* = 278)	35.9	47.7	10.566	0.005
CTX (*n* = 233)	36.4	29.5	8.265	0.016
MEM (*n* = 24)	3.3	3.6	0.168	0.919

Key: CIP, ciprofloxacin; CHL, chloramphenicol; NAL, nalidixic acid; GEN, gentamycin; AMP, ampicillin; TET, tetracycline; DOX, doxycycline; SXT, trimethoprim/sulfamethoxazole; CTX, cefotaxime; MEM, meropenem.

**Table 3:** Prevalence of antibiotic-resistant *E. coli* from poultry by ward.

No. of *E. coli* per Ward	% of Resistance to the Tested Antibiotic									
	CIP	CHL	NAL	GEN	AMP	TET	DOX	SXT	CTX	MEM
Ukonga (30)	56.7	43.3	73.3	22.4	70.0	61.2	59.7	49.3	63.3	3.0
Kipawa (67)	32.8	29.9	64.2	30.0	52.2	80.0	66.7	56.7	43.3	10.0
Gongolamboto (22)	50.0	36.4	72.7	31.8	36.4	81.8	63.6	54.5	68.2	0.0
Buguruni (15)	33.3	20.0	46.7	40.0	40	60.0	20.0	26.7	33.3	6.7
Kinyerezi (53)	39.6	39.6	56.6	50.9	50.9	64.2	43.4	41.5	39.6	5.7
Segerea (47)	34.0	31.9	63.8	34.0	53.2	72.3	46.8	55.3	61.7	4.3
Overall resistance	39.3	34.2	63.2	34.2	52.1	68.4	52.1	48.7	50.4	4.7
*p*-value	0.237	0.569	0.419	0.046	0.223	0.254	0.018	0.315	0.026	0.599

Key: CIP, ciprofloxacin; CHL, chloramphenicol; NAL, nalidixic acid; GEN, gentamycin; AMP, ampicillin; TET, tetracycline; DOX, doxycycline; SXT, trimethoprim/sulfamethoxazole; CTX, cefotaxime; MEM, meropenem.

**Table 4:** Prevalence of antibiotic-resistant *E. coli* from domestic pigs by ward.

No. of *E. coli* per Ward	% of Resistance to the Tested Antibiotic									
	CIP	CHL	NAL	GEN	AMP	TET	DOX	SXT	CTX	MEM
Ukonga (42)	23.8	21.4	33.3	19.0	27.8	50.0	27.8	33.3	42.9	5.6
Kipawa (18)	11.1	5.6	33.3	11.1	54.8	64.3	54.2	61.9	55.6	7.1
Kinyerezi (35)	34.3	25.7	37.1	25.7	54.3	51.4	37.1	62.9	20.0	0.0
Segerea (58)	24.1	22.4	43.1	22.4	48.3	53.4	41.4	41.4	27.6	1.7
Kisarawe (51)	60.8	62.7	64.7	47.1	76.5	80.4	60.8	80.4	47.1	11.8
Pugu station (18)	38.9	38.9	44.4	44.4	72.2	66.7	50.0	50.0	38.9	0.0
Overall resistance	34.2	32.0	44.6	28.8	57.2	62.2	46.8	57.7	36.7	5.0
*p*-value	0.000	0.000	0.031	0.006	0.003	0.033	0.101	0.000	0.034	0.022

Key: CIP, ciprofloxacin; CHL, chloramphenicol; NAL, nalidixic acid; GEN, gentamycin; AMP, ampicillin; TET, tetracycline; DOX, doxycycline; SXT, trimethoprim/sulfamethoxazole; CTX, cefotaxime; MEM, meropenem.

**Table 5:** Multidrug resistance patterns among 431 *E. coli* isolated from poultry and domestic pigs.

No. of Antibiotics Classes	Resistance Pattern	No. of Isolates	Prevalence (%)
3	TET/SUL/CEP	4	0.87
	QNL/PEN/SUL	6	1.30
	AMN/PEN/TET	3	0.65
	QNL/AMN/TET	8	1.73
	QNL/PEN/TET	3	0.65
	PEN/SUL/CEP	2	0.43
	QNL/PEN/TET	5	1.08
	PEN/TET/SUL	14	3.04
	QNL/TET/SUL	6	1.30
	PHE/TET/SUL	3	0.65
	QNL/TET/CEP	3	0.65
4	QNL/AMN/TET/CEP	5	1.08
	QNL/PEN/SUL/CEP	4	0.87
	QNL/AMN/TET/SUL	3	0.65
	QNL/PEN/TET/SUL	15	3.25
	PHE/QNL/PEN/TET	3	0.65
	PHE/PEN/TET/SUL	4	0.87
	PEN/TET/SUL/CEP	2	0.43
	QNL/TET/SUL/CEP	4	0.87
5	QNL/AMN/TET/SUL/CEP	3	0.65
	QNL/PEN/TET/SUL/CEP	5	1.08
	QNL/AMN/PEN/TET/SUL	4	0.87
	QNL/PHE/TET/SUL/CEP	3	0.65
	QNL/PHE/PEN/TET/SUL	16	3.47
6	QNL/AMN/PEN/TET/SUL/CEP	2	0.43
	QNL/PHE/AMN/PEN/TET/CEP	2	0.43
	QNL/PHE/PEN/TET/SUL/CEP	3	0.65
	QNL/PHE/AMN/PEN/TET/SUL	20	4.34
7	QNL/PHE/PEN/TET/SUL/CEP/CAR	11	2.39
	QNL/PHE/AMN/PEN/TET/SUL/CEP	62	13.45
8	QNL/PHE/AMN/PEN/TET/SUL/CEP/CAR	10	2.17
Total		238	51.6

Key: QNL, quinolones; PHE, phenocols; AMN, aminoglycosides; PEN, penicillins; TET, tetracyclines; SUL, sulfonamides; CEP, cephalosporins; CAR, carbapenems.

**Table 6:** Comparative antibiotic resistance of extended-spectrum beta lactamase (ESBL)- and non-ESBL-producing *E. coli* in cloacal and rectal swabs of poultry and domestic pigs.

Antibiotic	% of Resistant ESBL *E. coli* Producers (*n* = 301)	% of Resistant Non-ESBL *E. coli* Producers (*n* = 160)	*p*-Value
Ciprofloxacin	41.2(124)	30.6(49)	0.000
Chloramphenicol	36.2(109)	28.1(45)	0.000
Nalidixic acid	59.8(180)	45.0(72)	0.000
Gentamycin	33.6(101)	29.4(47)	0.000
Tetracycline	70.1(211)	57.5(92)	0.000
Doxycycline	52.5(158)	45.6(73)	0.000
Trimethoprim/ Sulfamethoxazole	55.5(167)	50.0(80)	0.000

**Table 7:** Comparative antibiotic resistance of quinolone-resistant versus non-quinolone-resistant *E. coli.*

Antibiotic	% Quinolone Resistance (*n* = 173)	% Non-Quinolone Resistance (*n* = 288)	Chi-Square	*p*-Value
Chloramphenicol	70.5(122)	11.1(32)	171.469	0.000
Gentamycin	60.1(104)	15.3(44)	99.683	0.000
Ampicillin	82.1(142)	37.2(107)	87.830	0.000
Tetracycline	92.5(160)	49.7(143)	88.022	0.000
Doxycycline	80.9(140)	31.6(91)	105.191	0.000
Trimethoprim/ Sulfamethoxazole	82.7(143)	36.1(104)	94.152	0.000
Cefotaxime	52.0(90)	40.6(117)	5.675	0.017
Meropenem	11.0(19)	1.0(3)	24.028	0.000

**Table 8:** Comparative results of ESBL producers against quinolone-resistant *E. coli* isolates.

ESBL Producer Isolate				Quinolone-Resistant Isolate			
	R	S	*p*-Value		R	S	*p*-Value
CHL	36.2(109)	63.8(192)	0.080	CHL	70.5(122)	11.1(32)	0.000
GEN	33.6(101)	64.4(200)	0.360	GEN	60.1(104)	15.3(44)	0.000
TET	70.1(211)	29.9(90)	0.007	TET	92.5(160)	49.7(143)	0.000
DOX	52.5(158)	47.5(143)	0.160	DOX	80.9(140)	31.6(91)	0.000
SXT	55.5(167)	44.5(134)	0.261	SXT	82.7(143)	36.1(104)	0.000

Key: CIP, ciprofloxacin; CHL, chloramphenicol; NAL, nalidixic acid; GEN, gentamycin; AMP, ampicillin; TET, tetracycline; DOX, doxycycline; SXT, trimethoprim/sulfamethoxazole; CTX, cefotaxime; MEM, meropenem.

**Table 9:** Distribution of ESBL and quinolone-resistant genes from the selected multidrug-resistant (MDR) *E. coli* (*n* = 20).

AMR Genes	*E. coli* No (%)	Sample Type	
		Poultry	Domestic Pigs
*bla*_CTX-M_	16/20 (80)	7	9
*bla*_TEM_	0/20 (0)	0	0
*bla*_SHV_	0/20 (0)	0	0
*qrnA*	0/20 (0)	0	0
*qrnB*	2/20 (10)	0	2
*qnrS*	0/20 (0)	0	0
*qnrC*	0/20 (0)	0	0
*qnrD*	0/20 (0)	0	0
*aac*(*6*)-*Ib*-*cr*	3/20 (15)	2	1
*qepA*	1/20 (5)	1	0

**Table 10:** PCR primers, sequences, and protocols used.

Gene	Primer Set and Sequence (5′-3′)	Amplicon Size	Reference
CTX-M	F: SCSATGTGCAGYACCAGTAA R: ACCAGAAYVAGCGGBGC	585 bp	[90,91]
*qnrA*	F: TCAGCAAGAGGATTTCTCA R: GGCAGCACTATTACTCCCA	627 bp	[92]
*qnrB*	F: GGMATHGAAATTCGCCACTG R: TTTGCYGYYCGCCAGTCGAA	264 bp	[92]
*qnrS*	F: ATGGAAACCTACAATCATAC R: AAAAACACCTCGACTTAAGT	467 bp	[92]
*aac*(*6*′)-*Ib*-*cr*	F: TTGCGATGCTCTATGAGTGGCTA R: CTCGAATGCCTGGCGTGTTT	482 bp	[92,93]
TEM	F: ATGAGTATTCAACATTTCCG R: CTGACAGTTACCAATGCTTA	868 bp	[94]
SHV	F: GGTTATGCGTTATATTCGCC R: TTAGCGTTGCCAGTGCTC	867 bp	[94]
*qnrC*	F: GGGTTGTACATTTATTGAATC R: TCCACTTTACGAGGTTCT	447 bp	[75]
*qnrD*	F: CGAGATCAATTTACGGGGAATA R: AACAAGCTGAAGCGCCTG	582 bp	[75]
*qepA*	F: TGGTCTACGCCATGGACCTCA R: TGAATTCGGACACCGTCTCCG	1137 bp	[75]

## Data Availability

The data generated is contained within the article.
